# Curative-intent ablation margins (A0) for colorectal liver metastasis: more burning questions

**DOI:** 10.1093/bjs/znae184

**Published:** 2024-08-30

**Authors:** Kjetil Søreide, Niels F M Kok

**Affiliations:** Department of Gastrointestinal Surgery, Stavanger University Hospital, Stavanger, Norway; Department of Clinical Medicine, University of Bergen, Bergen, Norway; Division of Surgery and Oncology, Department of Clinical Science, Intervention and Technology, Karolinska Institutet, Karolinska University Hospital, Stockholm, Sweden; Department of Surgical Oncology, Netherlands Cancer Institute, Amsterdam, the Netherlands

Almost half of all patients with colorectal cancer either present with, or eventually develop, metastases, and the liver is the most frequent location of disseminated disease. The standard of care for patients with colorectal liver metastasis (CRLM) has been surgical resection, whenever technically feasible and the patient deemed operable. Of note, surgical resection comes with the risk of associated morbidity, even though minimally invasive techniques (laparoscopic or robotically assisted), which facilitate faster recovery and are associated with a slightly lower risk of complications in selected patients, are increasingly being entertained. However, global dissemination of minimally invasive liver surgery is hampered by distribution, costs, and access to minimally invasive platforms, in particular for robotically assisted liver surgery. Furthermore, the simple use of size, numbers, and location of the CRLMs may not automatically justify the risk associated with open major liver resection, as there are other, less invasive alternatives to consider. For example, a small lesion located deep in the right hemiliver may be better treated locally with thermal ablation than by formal right hemihepatectomy. At least technically, an ablation will be associated with less morbidity and faster recovery for the patient. However, for treatment of CRLM with curative intent, the idea that ablative therapy is comparable to resection has been somewhat of a hard sell, with little robust evidence to support equipoise for oncological outcomes. Indeed, systematic reviews^1^ have shown that patients with CRLM treated with radiofrequency ablation (RFA) experienced fewer complications, but RFA was also associated with higher recurrence rates and poorer survival. Failure to completely destroy all cancerous cells in a lesion with ablation, and hence not obtain safe and adequate margins, has been stated as the likely reason for poorer oncological outcomes. As the tissue is not taken out (as in resection), the burning question has been how best to measure, monitor, and safely secure adequate control of the cancer lesion after ablation.

**Fig. 1:**
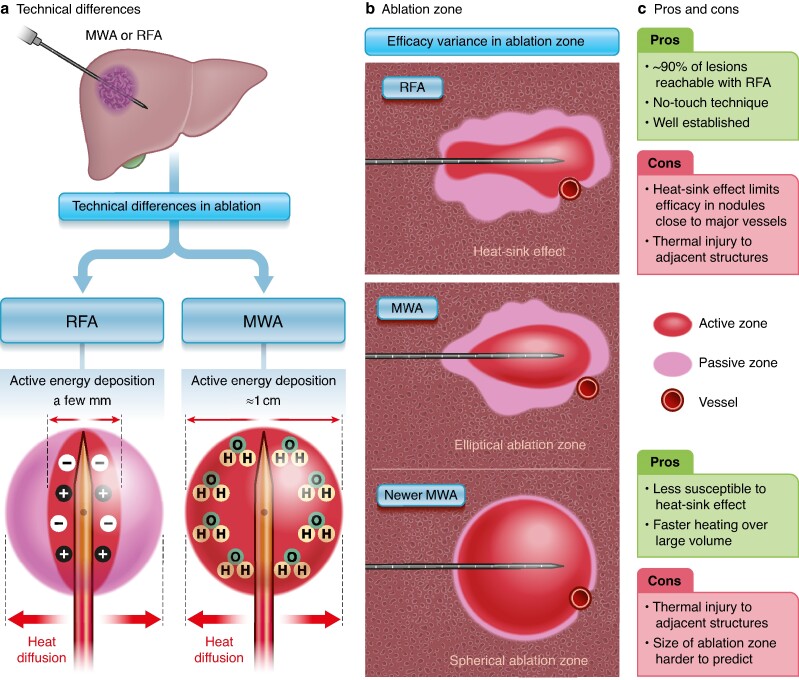
Ablation techniques and principles **a** Technical differences in radiofrequency ablation (RFA) and microwave ablation (MWA) techniques. **c** Resulting ablation zones, with active and passive fields and heat-sink effects resulting from vessels near ablation zone. **c** Pros and cons for each technique.

In open or minimally invasive liver resection, the resected specimen can be evaluated for radicality (R0) by histopathology under the microscope. One should note that the pathologist can only evaluate what is in the microscopic margin of the specimen offered. Surgical aspirators may theoretically remove a wider margin than is seen in the surface of the resected specimen, in particular for parenchyma-sparing procedures. Historically, a margin of at least 10 mm was believed to be necessary for radicality, but this has changed to 1 mm or more, as confirmed in recent studies^2^. Rather than margin width *per se*, it seems that tumour biology, molecular markers, and the growth pattern in tumour–liver interface are more important for actual oncological outcomes^3^. Hence, the view of radicality by margin evaluation, as achieved with either surgical resection over ablative techniques, has somewhat changed. In addition, the improvement in ablation technology (*Fig. 1*), with notable differences between radiofrequency and microwave ablative techniques, has led to more frequent use of ablation in some institutions, and also for resectable CRLM with curative intent or in combination with resection. A systematic review^4^ of available observational data on solitary, resectable lesions has suggested that disease-free and overall survival are better after liver resection than ablation for all lesion sizes, but RFA is likely to be comparable or non-inferior for lesions smaller than 3 cm. A prospective study (MAVERICC trial)^5^ with a matched control group from the Swedish Cancer Registry showed similar survival after thermal ablation of up to five lesions of 3 cm or smaller compared with liver resection. There does not seem to be a large difference between RFA and microwave ablation (MWA) techniques in local control, but MWA has the benefit of being faster and is associated with less heat-sink effect close to major vessels^6^ (*Fig. 1*).

**Fig. 2:**
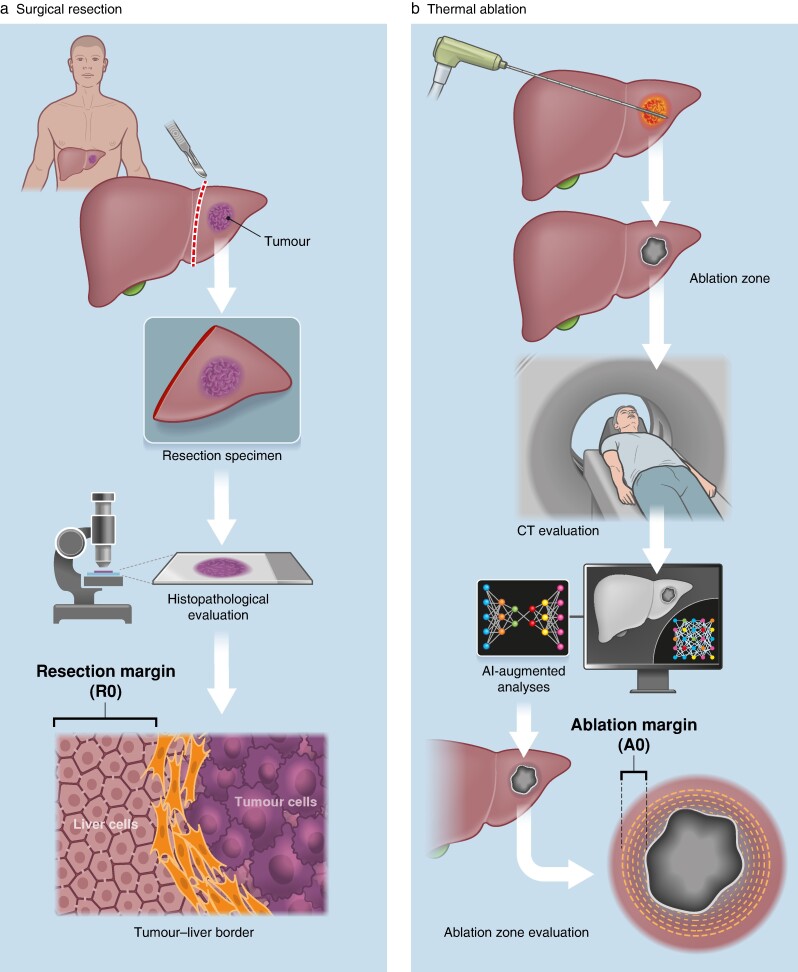
Comparison of radical resection margin (R0) and radical ablation margin (A0) Radicality **a** after surgical resection and **b** after thermal ablation. AI, artificial intelligence.

After liver resection, a radical resection margin (R0) may be determined by histopathological evaluation of the resection margin (*Fig. 2*). A similar evaluation of the ablation field has not been possible, and this has been one of the arguments against a safe and radical ablation zone that would translate into similar oncological outcomes. Furthermore, practice in postablation assessment has differed, with some undertaking control imaging immediately after the ablation (with the opportunity for additional ablation in the event of non-radicality) and others doing postablation monitoring for local recurrence. Independently of strategy, assessment of the radical ablation margin (called A0, similar to a surgical R0) is somewhat controversial, with a similar discussion evolving about radicality as has been entertained for resectional treatment of CRLM. Defining the appropriate ablation margin, and when and how this can best be assessed, this remain burning questions.

In this issue of *BJS*^7^, ablation margins associated with optimal oncological outcomes were investigated in a multicentre study. Several lessons can be learned from this analysis. The study exemplifies the variation between institutions in terms of patient selection, criteria used, and choice of technology. Hence, both the current and previous similar studies are prone to bias, yet may provide data for which no better experimental design currently exists. The study also shows how artificial intelligence can be incorporated into image-based assessment of the ablation zone (*Fig. 2*), and so help predict the safest minimal ablation margin, analogous to the histopathological resection margin for liver resections. Of note, however, almost one-third of all patients in this multicentre study had extrahepatic disease. This may call into question the role and importance of local disease control as a treatment with curative intent, and is largely dependent on how and to what intent the extrahepatic disease was treated. Hence, the definition and use of the A0 designation for the liver may have a role in determining local control, local disease progression, and local treatment completion, but less so for disease-free interval or overall survival. This should be kept in mind when interpreting the results, and the role of the stated A0 margin.

Several randomized trials have been initiated to investigate the effects of liver resection compared with thermal ablation. To date, only limited information can be obtained based on randomized trial data. In the multicentre LAVA trial^8^, high-risk (frail or elderly) patients were intentionally randomized to either resection or ablation, but the study was stopped early owing to lack of accrual. In the COLLISION trial^9^, randomization was conducted by target lesion. Patients were stratified into three groups depending on tumour burden, which could be up to 10 lesions no larger than 3 cm, with overall survival as primary outcome. After 299 of the anticipated 618 patients had been randomized, the trial was stopped because non-inferiority of thermal ablation compared with resection regarding the primary endpoint (overall survival) was very likely (HR 1.04, 95% c.i. 0.69 to 1.58; *P* = 0.846). Moreover, ablation resulted in fewer complications, a shorter hospital stay, and improved local control. Patients with up to five lesions no larger 3 cm are being recruited into the ongoing Scandinavian NewCOMET trial (NCT05129787). Two-hundred and thirty patients will be randomized to either thermal ablation only or resection only. The primary endpoint is local control, with results pending.

Thermal ablation is an essential tool for local treatment of CRLM. Old dogmas that resection is always better, and that ablation margins should always be extensive, should be left behind. Ablation is here to stay and liver surgeons should incorporate it into their decision-making. Surgeons should either embrace it as part of their own toolbox or have easy access to thermal ablation in the institution. There is no one-size-fits-all strategy in patients with CRLM, and the accumulated options and treatment pathways are multiple^10^. Larger CRLMs or lesions near main bile ducts may not be amenable to ablation. Ablation technology continues to develop, which will further boost its use. Newer techniques include use of intra-arterial contrast during the procedure, which is common during percutaneous procedures. It may reveal several other metastases before the first lesion is treated and lead to a change in management. Intraoperative navigation of vanishing lesions on imaging may also facilitate adequate treatment of CRLM with short thermal ablation^11^.
